# Similar enzymatic functions in distinct bioluminescence systems: evolutionary recruitment of sulfotransferases in ostracod light organs

**DOI:** 10.1098/rsbl.2023.0585

**Published:** 2024-05-15

**Authors:** Emily S. Lau, Jessica A. Goodheart, Nolan T. Anderson, Vannie L. Liu, Arnab Mukherjee, Todd H. Oakley

**Affiliations:** ^1^ Department of Ecology, Evolution, and Marine Biology, University of California Santa Barbara, Santa Barbara, CA 93106, USA; ^2^ Department of Chemical Engineering, University of California Santa Barbara, Santa Barbara, CA 93106, USA; ^3^ Department of Biological Engineering, University of California Santa Barbara, Santa Barbara, CA 93106, USA; ^4^ Department of Chemistry, University of California Santa Barbara, Santa Barbara, CA 93106, USA; ^5^ Neuroscience Research Institute, University of California Santa Barbara, Santa Barbara, CA 93106, USA; ^6^ Division of Invertebrate Zoology, American Museum of Natural History, New York, NY 10025, USA

**Keywords:** convergent evolution, parallel evolution, complex traits, gene expression, bioluminescence, sulfotransferase

## Abstract

Genes from ancient families are sometimes involved in the convergent evolutionary origins of similar traits, even across vast phylogenetic distances. Sulfotransferases are an ancient family of enzymes that transfer sulfate from a donor to a wide variety of substrates, including probable roles in some bioluminescence systems. Here, we demonstrate multiple sulfotransferases, highly expressed in light organs of the bioluminescent ostracod *Vargula tsujii*, transfer sulfate *in vitro* to the luciferin substrate, vargulin. We find luciferin sulfotransferases (LSTs) of ostracods are not orthologous to known LSTs of fireflies or sea pansies; animals with distinct and convergently evolved bioluminescence systems compared to ostracods. Therefore, distantly related sulfotransferases were independently recruited at least three times, leading to parallel evolution of luciferin metabolism in three highly diverged organisms. Reuse of homologous genes is surprising in these bioluminescence systems because the other components, including luciferins and luciferases, are completely distinct. Whether convergently evolved traits incorporate ancient genes with similar functions or instead use distinct, often newer, genes may be constrained by how many genetic solutions exist for a particular function. When fewer solutions exist, as in genetic sulfation of small molecules, evolution may be more constrained to use the same genes time and again.

## 1. Introduction

The convergent evolutionary origins of similar traits sometimes employ existing genetic elements that originated much earlier. This pattern of parallel evolution is often called ‘deep homology’, especially when similar but convergently evolved traits express shared transcription factors. For example, limbs in some distantly related phyla express the transcription factor *distalless* [1]. Convergently evolved traits also may recruit ancient genes with shared enzymatic functions. For example, convergently evolved instances of biomineralization use α-carbonic anhydrases, an ancient gene family found across animals [2] that facilitates conversion of carbon dioxide to bicarbonate, crucial in regulating pH and mineral deposition. Similarly, convergently evolved cases of photosymbiosis involve vacuolar H^+^-ATPase (*VHA*), another ancient gene family [3,4], which plays a vital role to acidify intracellular compartments, essential for nutrient transport and cellular metabolism. Finally, convergently evolved cases of bioluminescence may have recruited ancient dehalogenases [5,6]. Characterizing new examples where homology differs across levels of biological organization will build the knowledge necessary to answer fundamental evolutionary questions, such as what types of traits, genes or evolutionary histories are more likely to lead to the use of ancient elements in novel systems (e.g. [7]).

We hypothesize sulfotransferases, a family of enzymes shared across vast phylogenetic distances, are used in convergently evolved bioluminescence systems of distantly related species. Fireflies, sea pansies and ostracods convergently evolved bioluminescence systems and produce light using structurally diverse organs or tissues [8–10], different luciferin substrates [11], and non-homologous, autogenic, luciferase enzymes [12,13] (electronic supplementary material, figure S1). Despite these vast differences, these taxa use the same biochemical mechanism, sulfation—the transfer of sulfate (SO_3_^−^) from the donor 3′-phosphoadenosine-5′-phosphosulfate (PAPS) to a substrate [14]—to modulate the chemical state of luciferins [15–18] (electronic supplementary material, figure S2). Sulfation may create chemical forms of the substrate that are better for storage by making them less susceptible to non-specific oxidation [16–19], or may create an activated form of the substrate for the luciferase reaction [20]. Sulfation is catalysed by luciferin sulfotransferases (LSTs) in fireflies [17] and sea pansies [18], but less is known about ostracod sulfotransferases. Although previously published results do show crude protein extracts from the ostracod *Vargula hilgendorfii* can reversibly sulfate luciferin, thereby suggesting sulfation as a mechanism for luciferin metabolism in the organism [16], the genetic sequences of LSTs in ostracods have yet to be identified and functionally characterized.

Here, we provide evidence of independent recruitment of paralogous genes from a single, ancient gene family—sulfotransferases—leading to parallel evolution of luciferin sulfation in phylogenetically distant organisms with distinct bioluminescence systems. We use gene expression analyses, recombinant protein expression, and *in vitro* biochemical assays to identify and functionally test the five most highly expressed sulfotransferase genes from the light-producing organ of the cypridinid ostracod *V. tsujii* and report multiple sulfotransferases capable of sulfating vargulin *in vitro*. Our results, taken together with functional evidence of LSTs previously published on sea pansies and fireflies [17,18] provide an example of an ancient and pervasive gene family that was recruited multiple times independently for a similar purpose.

## 2. Material and methods

### (a) Reference transcriptome assembly for *Vargula tsujii*

We constructed a reference transcriptome for *V. tsujii* by pooling RNA from 88 specimens across various instars (see electronic supplementary material, table S1 and figures S3 and S4A). We extracted RNA using TRIzol^®^ for sequencing at the BYU Sequencing Centre using PacBio Sequel II (IsoSeq) using two transcript size fractions: 4–10 kb selected using BluePippin (Sage Science) and a non-size-selected fraction. Next, we processed circular consensus sequencing reads with the IsoSeq v3 pipeline (https://github.com/PacificBiosciences/IsoSeq) and combined them with Illumina short reads (SRR21201581, [21]) using rnaSPAdes with flags –rna, –only-assembler and –trusted-contigs [22]. We clustered sequences with ≥ 95% identity using cd-hit-est [23,24].

### (b) Sulfotransferase candidates from the upper lip of *Vargula tsujii*

To quantify gene expression, we collected RNA from light organs (upper lips) of five male and four female ostracods maintained in culture [25] (electronic supplementary material, figure S4B). After extracting RNA with Trizol, we used the Genomic Sequencing and Analysis Facility at UT-Austin for library preparations and Tag-Seq profiling using Illumina HiSeq 2500, SR100 [26]. We processed Tag-Seq reads (https://github.com/z0on/tag-based_RNAseq) by mapping to our reference transcriptome with Bowtie2 v. 2.3.4.3 [27] (electronic supplementary material, table S2). For protein sequence identification, we translated sequences with TransDecoder v. 5.5.0 and used HMMER v. 3.2.1 (hmmer.org) to find complete protein sequences containing a sulfotransferase domain (Pfam ID: PF00685) of e-value ≤ 1 × 10^−3^.

### (c) Cloning, expression and purification of *Vargula tsujii* sulfotransferase candidates

We synthesized DNA sequences for candidate LSTs ST1–5 (electronic supplementary material, figure S5) as gBlocks (Integrated DNA Technologies) and cloned them into bacterial expression vectors (ST3 and ST4 into pQE80L; ST1, ST2 and ST5 in pET SUMO to improve solubility [28] (electronic supplementary material, figures S6 and S7) using Gibson assembly (electronic supplementary material, table S3). These vectors allow protein expression with N-terminal hexahistidine tags for purification using metal-affinity chromatography. We propagated plasmid constructs in *E. coli* NEB 10-beta cells and verified by Sanger sequencing (Genewiz, South Plainfield, NJ, USA).

We expressed proteins in *E. coli* BL21 cells via induction with IPTG, followed by incubating cultures at room temperature for 18 h. We purified proteins by incubating lysed cell extracts with Ni-NTA agarose beads, then using gravity-flow metal-affinity chromatography to elute hexahistidine-tagged proteins. We concentrated eluates by spin filtration (10 kDa molecular weight cut-off) and assessed protein purity via SDS-PAGE (electronic supplementary material, figure S7 and table S4) and densitometry. We quantified purified protein yields using a Bradford assay (Bio-Rad Laboratories, Hercules, CA, USA) calibrated with a bovine serum albumin standard. These methods successfully purified all five proteins with > 90% homogeneity (electronic supplementary material, table S5), and we prepared proteins freshly for each experiment.

### (d) Sulfotransferase functional assays

We tested for sulfotransferase activity using two functional assays. First, we used a commercially available sulfotransferase assay (R&D Systems, Minneapolis, MN, USA) that probes for the formation of 3′-phosphoadenosine-5′-phosphate (PAP), a by-product of sulfation, by measuring the increase in absorbance at 620 nm following the enzymatic hydrolysis of PAP into phosphate, which binds to malachite green. In this assay, the phosphatase used to hydrolyse PAP into phosphate has some activity with PAPS, so we used an enzyme-free negative control and kept PAPS the same between experiments and control.

The second assay quantifies decreases in luminescence to test each candidate protein's ability to convert luciferin to its sulfated form, which luciferase does not oxidize. For ostracod luciferase, we transfected CHO cells with a pCMV *Cypridina* luciferase vector, and purified the protein with metal-affinity chromatography (electronic supplementary material, figure S8). We first mixed each purified protein with ostracod luciferin and PAPS, incubated at 25°C for 3 h, then quenched reactions by heat inactivation. After cooling, we added the diluted reaction product to purified ostracod luciferase and immediately measured bioluminescence. We compared luminescence between test and enzyme-free reactions with a decrease in light emission indicating less luciferin available for light production due to conversion to its sulfated form. In addition to luciferin sulfation assays, we used an established p-nitrophenol sulfation assay to evaluate sulfotransferase candidates for their ability to sulfate p-nitrophenol, a common substrate for many sulfotransferases, including those unrelated to bioluminescence (electronic supplementary material, figure S9).

### (e) Phylogenetic analyses

We estimated a phylogenetic tree for sulfotransferase candidates from *V. tsujii* and a tree of functionally tested sulfotransferases from the literature (as of March 2023) (electronic supplementary material, table S6 and references). For aligning protein sequences, we used MAFFT (v. 7.429) [29], trimmed the alignment of functionally tested sulfotransferases using trimAl (v. 1.4) [30] with the flag-gappyout, inferred maximum-likelihood phylogenies using IQ-TREE (v. 1.6.12) [31], assuming the best-fit substitution model (LG + I + G4 for candidates, LG + F + R5 for functionally tested, LG + R5 for trimmed alignment of functionally tested) according to Bayesian information criterion, as determined with ModelFinder [32], and performed ultrafast bootstrap approximation [33] with 1000 bootstrap replicates. To midpoint root the phylogeny and visualize bootstrap values and expression values, we used the R packages phytools (v. 1.5-1) [34] and ggtree (v. 3.9.0) [35].

## 3. Results

### (a) Functional assays of sulfotransferase genes from the light organ of *Vargula tsujii*

**Figure 1.**
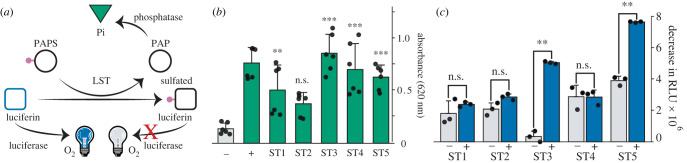
Based on two different assays, multiple candidate sulfotransferases from *Vargula tsujii*, especially ST3 and ST5, exhibit luciferin sulfation activity. (*a*) Although mass spectroscopy would be the most direct demonstration of vargulin sulfation, we illustrate a schematic of the chemistry underlying the two assays we use here. (*b*) Results of the absorbance-based sulfotransferase assay that probes for the conversion of PAPS (sulfate donor) to PAP. Our positive (+) control used 50 µl of 10 ng µl^−1^ Golgi-resident PAP-specific 3′ phosphatase and 50 µM PAP to confirm phosphatase activity and a negative (–) control used 50 µl of 10 ng µl^−1^ coupling phosphatase, 100 µM luciferin, 200 µM lithium-free PAPS, but lacks any sulfotransferase. The test reactions used 50 µl of 10 ng µl^−1^ coupling phosphatase, 100 µM luciferin, and 200 µM lithium-free PAPS, and 10 µM sulfotransferase. Three genes showed the highest activity (ST3–5) and ST1 also showed significant activity under these conditions. (*c*) Results of the bioluminescence assay that probes for a decrease in light emission due to sulfation of luciferin, which decreases luciferin available for oxidation. Negative control reactions (grey bars) lack PAPS. Test reactions (blue bars) included 10 µl of 10 µM ostracod luciferin and 100 µM PAPS with 1 µl of each purified protein (final concentrations ST1: 15 µM, ST2: 22 µM, ST3: 51 µM, ST4: 32 µM, ST5: 25 µM). Variations across the negative controls likely arise from the spontaneous oxidation of luciferin. Compared to control PAPS-free reactions, two candidates (ST3, ST5) exhibit a significant decrease in light production indicative of sulfation activity sufficient to modulate bioluminescence *in vitro*. ST2 may have some activity, but was not statistically significant with Welch's test.

From Tag-seq on light-producing organs of *V. tsujii* (*N* = 9), we identified 40 genes, expressed in at least one individual, that contain domains with significant similarity to sulfotransferase domains, with nine expressed in all individuals. We selected the five (ST1-5) most highly expressed candidate sulfotransferases in light organs (electronic supplementary material, figure S10) for recombinant expression and performed two functional assays to test for luciferin (vargulin) sulfation activity (figure 1*b,c*). Based on the commercial absorbance-based assay that probes for sulfotransferase-dependent conversion of PAPS to PAP, we found three candidates have the highest fold-change (five- to sixfold) in activity (ST3, ST4 and ST5) with ST1 also showing statistically significant change in absorbance under the conditions used (figure 1*b*). Based on the bioluminescence assay, which measures the decrease in light emission caused by sulfating luciferin, we infer the largest magnitude of luciferin sulfation in ST3 and ST5 (figure 1*c*).

### (b) Ostracods, fireflies and sea pansies sulfate luciferin by using homologous genes

**Figure 2.**
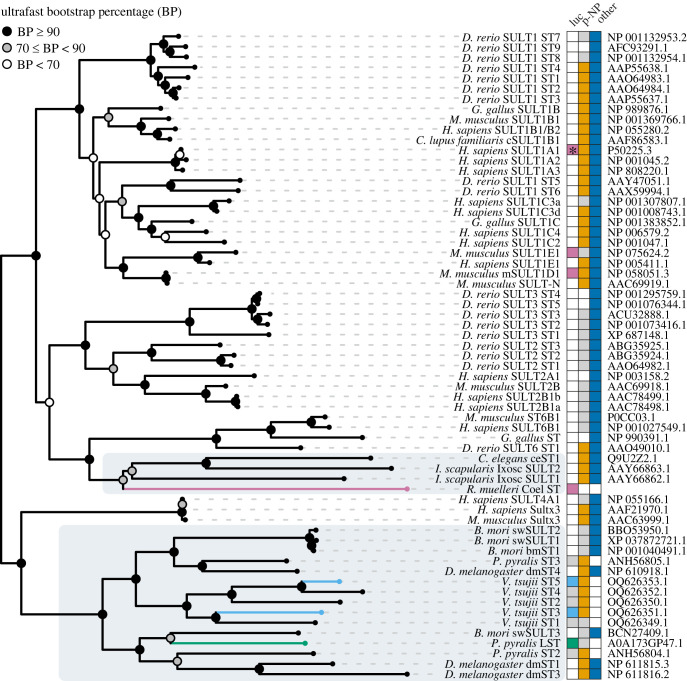
Maximum-likelihood phylogeny of functionally tested sulfotransferases. Luciferin sulfotransferases from luminous organisms are denoted by coloured branches (firefly = green, ostracods = light blue, sea pansies = purple). Invertebrate sulfotransferases are highlighted in grey. Coloured squares (green = firefly luciferin, light blue = ostracod luciferin for genes with strong activity in both assays herein, purple = sea pansy luciferin, orange = p-nitrophenol, dark blue = other) indicate functionally demonstrated substrates for each sulfotransferase; grey squares indicate molecules that are not sulfated by the respective sulfotransferase; white squares are untested. *Sulfates the luciferin coelenterazine at a different site compared to sea pansy sulfated luciferin [36]. Accession numbers are listed on the right. Ultrafast bootstrap values are represented by coloured circles at each node.

Genes of ostracods, fireflies and sea pansies convergently evolved sulfation capabilities (figure 2), probably independently recruiting sulfotransferase genes as a mechanism for metabolizing luciferin. Our phylogenetic analysis of functionally characterized LSTs are most closely related to sulfotransferases of non-luminous animals. We find firefly LST to be most closely related to a gene from a non-luminous silk moth *Bombyx mori* that sulfates multiple substrates. The LST of the sea pansy *R. muelleri* is most closely related to sulfotransferase genes from a non-luminous tick (*Ixodes scapularis*) and a nematode (*Caenorhabditis elegans*). The five candidate LSTs from the ostracod *V. tsujii* we tested form a clade sister to genes from non-luminous *B. mori*, and *D. melanogaster*, and ST3 from firefly, which does not sulfate luciferin [17]. The close relationship between LSTs to genes from non-luminous animals or genes lacking the ability to sulfate luciferin, strongly support convergent recruitment of LSTs in fireflies, sea pansies and ostracods (figure 2; electronic supplementary material, figure S11).

## 4. Discussion

Convergently evolved traits may independently deploy members of deeply conserved gene families, a pattern called deep homology [1]. Although criticized for imprecision and incompleteness [37,38], the term deep homology highlights the importance of ancient evolutionary histories in shaping new traits [39]. Here, we report sulfation in bioluminescence systems may have evolved by independently recruiting homologous genes from the ancient sulfotransferase gene family present across animals. We provide functional evidence for multiple sulfotransferases capable of sulfating luciferin in the ostracod *V. tsujii* and synthesize our results with those previously described in fireflies and sea pansies.

Although establishing the genetic basis of organismal functions requires gene knockdowns, multiple lines of evidence point to sulfotransferase involvement in the bioluminescence of ostracods. Our results demonstrate multiple sulfotransferase genes of *V. tsujii* catalyse a reaction to sulfate luciferin, but determining how many using statistical significance depends on the assay (figure 1). The commercial assay that probes for PAP indicates four genes with statistically significant sulfation of luciferin (with ST3–5 showing especially strong differences between test and control) and the ‘bioluminescence assay’ that probes directly for luciferin sulfation indicates two genes (ST3 and ST5). The statistical difference between assays is likely due to specific experimental conditions, with the bioluminescence assay requiring a lower concentration of luciferin (10 µM) than the absorbance assay (100 µM), due to substrate inhibition of luciferase (electronic supplementary material, figure S12). Nevertheless, results from both assays support multiple sulfotransferases highly expressed in the light organ—especially ST3 and ST5—are capable of sulfating luciferin. Forthcoming work indicates ST3 is the only sulfotransferase with significant co-expression with luciferase across multiple stages and tissues [40]. Based on this co-expression with luciferase, we speculate ST3 could be the primary sulfotransferase used in bioluminescence. Coupled with published results from crude protein extracts of *V. hilgendorfii* that sulfate luciferin [16], we hypothesize ST3 and possibly at least one other sulfotransferase (ST5) have important organismal functions in luminous ostracods, perhaps for use as a storage form of the easily oxidized luciferin.

A similar confluence of evidence suggests sulfotransferases are important to bioluminescence systems of fireflies and sea pansies. In fireflies, one sulfotransferase, highly expressed in lanterns, can catalyse formation of sulfoluciferin from luciferin and the reverse reaction [17]. This suggests an organismal function for an LST in fireflies, again perhaps for creating a storage form of the substrate [17]. In the anthozoan *R. muelleri*, sulfated luciferin is converted into luciferin, bound to a luciferin-binding protein, and released upon addition of calcium to react with luciferase [41]. Recently, two sulfotransferase isoforms, similar in sequence to the native protein, were cloned, expressed, and shown to have sulfotransferase activity [18]. Because preparations from *Renilla* yield much higher amounts of luciferin sulfate compared to the amount of luciferin bound to luciferin-binding proteins [42], the sulfated form probably acts as a storage form of the luminous substrate in the animal [43]. Therefore, despite difficulties in knocking down specific sulfotransferases in ostracods, fireflies or sea pansies, the combination of native preparations, gene expression studies and heterologous expression suggests these animals use sulfotransferases (electronic supplementary material, figure S13) in their respective bioluminescence systems.

Despite a growing realization of deep homology in evolutionary history, discovering homologous components in bioluminescence systems is arresting because of the distinctness of the other components. First, even the small-molecule substrates catalysed by sulfotransferases are different: the luciferin of ostracods is vargulin, fireflies is d-luciferin, and sea pansy is coelenterazine. The luciferases also evolved from non-homologous gene families [12,13,44]. At the organismal level, bioluminescence is created by very different structures. Ostracod bioluminescence is secreted outside the body by the upper lip [45], firefly light is created in an abdominal structure called the lantern, and in sea pansies, light production occurs in specific cells called photocytes, located in the endoderm of two types of polyps. The presence of homologous sulfotransferases with similar functions along with non-homologous luciferases and distinct substrates provides a striking example of the often varied, cobbled-together elements of evolved complex systems. As more examples accumulate of cases of ancient genes independently recruited for similar functions, a logical pattern begins to emerge. Deeply homologous genes tend to have generally useful functions, such as sulfating small molecules in the case of sulfotransferases. Especially when there are few other solutions—as there is no other demonstrated mechanism for sulfation besides sulfotransferases—evolution may recruit the same gene family time and again, even in animals as distantly related as cnidarians and arthropods. This highlights how constraints—in this case determined by the number of biological solutions—may bias the options available to evolution [46,47].

## Data Availability

All raw sequencing data are available on NCBI (BioProject: PRJNA935772). Sulfotransferase sequences are available on GenBank (accessions: OQ626349, OQ626350, OQ626351, OQ626352 and OQ626353). Plasmid sequences, code for analysing and visualizing data, and data files for gene expression, phylogenetic analyses and functional assays are available on Dryad (https://doi.org/10.25349/D94W5N) [48]. Supplementary material is available online [49].
